# Oscillations in continuous culture populations of *Streptococcus pneumoniae*: population dynamics and the evolution of clonal suicide

**DOI:** 10.1098/rspb.2008.1415

**Published:** 2008-12-03

**Authors:** Omar E. Cornejo, Daniel E. Rozen, Robert M. May, Bruce R. Levin

**Affiliations:** 1 Department of Biology, Emory UniversityAtlanta, GA 30322, USA; 2 Department of Life Sciences, University of ManchesterManchester M13 9PL, UK; 3 Department of Zoology, University of OxfordOxford OX1 3PS, UK

**Keywords:** evolution, chemostats, *Streptococcus pneumoniae*, allelopathy, population dynamics, mathematical models

## Abstract

Agents that kill or induce suicide in the organisms that produce them or other individuals of the same genotype are intriguing puzzles for ecologists and evolutionary biologists. When those organisms are pathogenic bacteria, these suicidal toxins have the added appeal as candidates for the development of narrow spectrum antibiotics to kill the pathogens that produce them. We show that when clinical as well as laboratory strains of *Streptococcus pneumoniae* are maintained in continuous culture (chemostats), their densities oscillate by as much as five orders of magnitude with an apparently constant period. This dynamic, which is unanticipated for single clones of bacteria in chemostats, can be attributed to population-wide die-offs and recoveries. Using a combination of mathematical models and experiments with *S. pneumoniae*, we present evidence that these die-offs can be attributed to the autocatalytic production of a toxin that lyses or induces autolysis in members of the clone that produces it. This toxin, which our evidence indicates is a protein, appears to be novel; *S. pneumoniae* genetic constructs knocked out for *lytA* and other genes coding for known candidates for this agent oscillate in chemostat culture. Since this toxin lyses different strains of *S. pneumoniae* as well as other closely related species of *Streptococcus*, we propose that its ecological role is as an allelopathic agent. Using a mathematical model, we explore the conditions under which toxins that kill members of the same clone that produces them can prevent established populations from invasion by different strains of the same or other species. We postulate that the production of the toxin observed here as well as other bacteria-produced toxins that kill members of the same genotype, ‘clonal suicide’, evolved and are maintained to prevent colonization of established populations by different strains of the same and closely related species.

## 1. Introduction

From an ecological and evolutionary perspective, it is not at all surprising that bacteria produce toxic agents that kill other species (Wiener 1996) or genetically different clones of the same species (Riley & Gordon 1999; Czaran *et al*. 2002; Riley & Wertz 2002). More interesting evolutionarily are the toxins produced by bacteria that kill or induce suicide (apoptosis and autolysis) in genetically identical members of their own species. Included among these agents are the murein hydrolases and the choline-binding proteins responsible for autolysis and allolysis in *Streptococcus pneumoniae* (Steinmoen *et al*. 2002, 2003; Moscoso & Claverys 2004; Guiral *et al*. 2005), and the proteins produced by sporulation-committed *Bacillus subtilis* that kill non-committed members of the same genotype (Gonzalez-Pastor *et al*. 2003; Ellermeier *et al*. 2006; Claverys & Havarstein 2007).

Three general classes of hypotheses have been presented for the selection pressures responsible for the evolution and maintenance of mechanisms that are lethal to members of the same genotype. One has been likened to cannibalism, where the killed cells provide nutrients which in times of dearth enable the population at large to survive (Claverys & Havarstein 2007). A variant of this cannibalism hypothesis has been proposed for *B. subtilis*, wherein the nutrients released by killed cells enable the remaining ‘killer’ cells to postpone the terminal commitment to sporulation, an energetically costly process that delays vegetative growth until new resources become available (Gonzalez-Pastor *et al*. 2003). In the second class of hypothesis, gastronomy is not the reason for killing one's own, but rather the acquisition of genetic information via transformation (a more intellectual goal?). In accord with this hypothesis, killing members of one's own genotype is coincidental to the lysis of bacteria of the same or related species which may carry genes that provide an advantage to the killer population, perhaps by enabling them to invade new habitats or survive environmental stresses such as antibiotics (Claverys & Havarstein 2007; Claverys *et al*. 2007). The third class of hypothesis of which we are aware considers lysis to be a stress response, which promotes the release of virulence factors (Pinas *et al*. 2008). Presumably, these factors would facilitate the ability of the surviving members of the killer population to colonize susceptible hosts.

In this paper, we present evidence for a new mechanism by which bacteria kill members of their same genotype that we discovered serendipitously. While performing experiments to estimate the rate constant of recombination (Levin 1981) in chemostat populations of *S. pneumoniae* (pneumococcus) strain R6, we observed oscillations in density of up to five orders of magnitude with an apparently constant period. These oscillations were not restricted to this domesticated laboratory strain but it was also obtained for the three clinical isolates of pneumococcus we examined. Although oscillations of substantial amplitude in the density of bacteria are expected when predators such as lytic bacteriophage are present (Chao *et al*. 1977; Yoshida *et al*. 2007), they are not anticipated in monoclonal cultures of bacteria free of a third trophic level of organisms. Under these conditions, bacteria are expected to maintain a constant density, the magnitude of which depends primarily on the concentration of the limiting resource (Monod 1949; Kubitzchek 1970; Stewart & Levin 1973).

Our theoretical and experimental analysis indicates that these oscillations in *S. pneumoniae* R6 can be attributed to the waxing and waning in the concentration of an agent (a toxin) that is released by the bacteria via an autocatalytic process and either lyses cells directly or induces their autolysis. Our experiments indicate that the toxin responsible for these oscillations is a protein that can also lyse or induce lysis in closely but not distantly related species of *Streptococcus*. Since we can rule out the six known suspects anticipated for this lysis, we conjecture that this oscillation-driving toxin is novel, i.e. has not been previously identified or characterized.

We present and, with the aid of a mathematical model, explore the properties of a fourth hypothesis for the evolution and maintenance of the observed, and other, toxins that kill members of the same clone: allelopathy to prevent the invasion of established populations by competing strains of the same and different species. We briefly discuss why resistance to the suicidal agent observed here has not evolved and the potential for using these toxins for the treatment of bacterial infections.

## 2. Material and methods

### (a) Bacteria

The stains and species of bacteria used in this investigation, their genetic markers and their source are listed in table 1.

### (b) Culture media

#### (i) Liquid

THY (per litre): 30 g Todd–Hewitt broth (Difco) plus 5 g yeast extract (Difco).

#### (ii) Solid (agar) for sampling

TSA: tryptic soy agar (Fisher) with 5 per cent sheep blood (Difco) and 16 g agar per litre.

MHA: Mueller–Hinton base (Difco) with 16 g agar per litre.

### (c) Chemostat culture and sampling

The chemostats used in these experiments are ‘home-made’ (see www.eclf.net for the design and photographs). Chemostat populations were established from −80°C freezer stocks of *S. pneumoniae* via a two-step process: 200 μl aliquots of frozen cells were thawed in 2 ml of THY and grown to an OD_600_∼0.3 (corresponding to a density of approximately 2e^8^ cells ml^−1^) at which time they were diluted 10-fold in THY broth and regrown to OD_600_∼0.3. Aliquots of 100 μl of these regrown cultures were inoculated into 20 ml of THY broth in the chemostat vessels for an initial density of approximately 1×10^6^ cells ml^−1^. The chemostat vessels (tubes) were incubated at 35°C in a water bath. A peristaltic pump maintained a constant flow of fresh media at a rate of 2 ml per hour, which is the same rate at which the waste, excess resources and live or dead cells were removed from the vessel. The chemostat cultures were agitated and aerated by a vacuum-induced bubbling of filter-sterilized air. The samples were taken by removing approximately 500 μl directly from the chemostat vessel. Viable cell densities (CFU) were estimated by serially diluting the samples in 0.85 per cent saline and plating on tryptic soy agar containing 5 per cent sheep blood. The experiments with low densities of *S. pneumoniae* were performed in chemostats with a reservoir containing THY and 0.85 per cent saline in a ratio of 1 : 28.

### (d) Bioassay for toxin

To test for the presence and relative concentration of a toxin, samples taken at different times were centrifuged for 2 min at 13 000*g*, and then the supernatant was stored at −20°C. No CFUs were observed when 100 μl samples of the centrifuged supernatants were plated on tryptic soy agar with 5 per cent sheep blood, indicating that the supernatants were cell free. Between 1.5 and 2.0 μl of these supernatants were spotted onto soft-agar THY lawns of target bacteria on a base of Mueller–Hinton (hard) agar. These lawns were prepared by growing *S. pneumoniae* R6 or other strains of this or other species of streptococci in THY to an OD_600_∼0.3 nm and adding 100 μl to 4 ml of soft agar at approximately 55°C (approx. 5×10^6^ cells ml^−1^). The plates containing the supernatant-spotted lawns were scored after 24 hours of incubation at 35°C in a CO_2_ incubator. To test whether the zones of inhibition on the agar were due to replicating phage, the material from zones of inhibition was added to fresh soft agar with *S. pneumoniae* and transferred to new lawns. No plaques were observed.

To ascertain whether the toxin was hydrogen peroxide (H_2_O_2_), we added approximately 5000 units of catalase (Worthington Biochemical) to 3 ml of soft agar used for the lawns, or incubated the supernatants from the chemostats for 30 min with 5000 units catalase before spotting. To determine whether the toxin had a protein nature, we incubated the supernatant at 55, 65 and 75°C for 30 min before assaying its activity. Also, prior to assaying for activity, the supernatant was treated for 30 min at 37°C with proteinase K at a final concentration of 1.5 mg ml^−1^. Initial screening to determine the size range of the putative protein toxin was performed using membrane centrifugal filters (Microcon^TM^) with cut-offs of 3, 10, 30 and 50 kDa following the manufacturer's protocols.

### (e) Numerical analysis (simulations)

Numerical solutions to the differential equations of our models (simulations) were programmed in R (a free software environment for statistical computing) and/or Berkeley Madonna. The codes for these simulations are available on www.eclf.net.

## 3. Results

**Figure 1:**
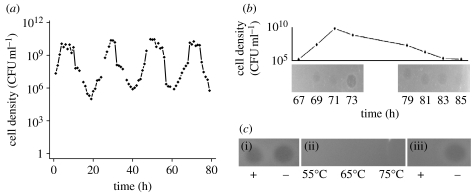
*Streptococcus pneumoniae* R6 in chemostat culture. (*a*) Cell density estimated from colony-forming units (CFU ml^−1^). (*b*) Supernatant taken at different times during the course of a cycle spotted onto soft-agar lawns of exponentially growing *S. pneumoniae* R6. (*c*) (i) Catalase, (ii) heat-treated and (iii) proteinase K (protease)-treated supernatants on lawns of exponentially growing *S. pneumoniae* R6. Unless otherwise noted, this and the other chemostats in this study were maintained at a dilution rate of approximately 0.1 per hour in the Todd–Hewitt broth plus 0.5% yeast extract (THY).

**Figure 2:**
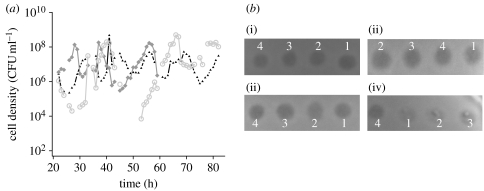
(*a*) Clinical isolates show oscillatory dynamics that qualitatively resemble those obtained with the laboratory strains (black triangles, PMEN-18; grey squares, PMEN-6; grey circles, PMEN-20). The source of these and other strains used in this study are listed in table 1. (*b*) Cross activity of supernatants among different strains. Spot assays performed on lawns (backgrounds) of the laboratory strain R6, and three clinical isolates (PMEN-18, PMEN-6 and PMEN-20). The supernatant from single-clone chemostats of *S. pneumoniae*: (i) R6 (1), (ii) PMEN-6 (4), (iii) PMEN-20 (2) and (iv) PMEN-18 (3), and tested on lawns of each cell line.

We open this paper with the serendipitous observation that motivated this investigation, profound oscillations in the density of *S. pneumoniae* R6 in a chemostat (figure 1*a*). Similar observations were obtained with all three clinical isolates of *S. pneumoniae* that we examined (figure 2*a*). These dramatic oscillations in colony-forming units (CFU) are also apparent from changes in the optical density (OD_620_ nm) in these cultures. If these bacteria were killed but not did lyse, the OD of the chemostat would be expected to roughly halve every 6.9 hours at the flow rate employed (0.1 per hour) and the culture would remain turbid for more than 24 hours. Instead, we observed a complete loss of turbidity over roughly 10 hours, which we interpret as evidence that the decline in the CFU estimates of density in these chemostats can be attributed to the bacteria lysing.

By spotting the supernatants of centrifuged samples on soft-agar lawns of *S. pneumoniae* R6 (see §2), we assayed for the presence of an exogenous agent released by the bacteria, a ‘toxin’, that could account for the declines in the densities. The results of these assays were positive; zones of inhibition were observed (figure 1*b*). Based on the clarity of the zone, the concentration of this toxin appears to reach an apex shortly after the bacteria reach their maximum density (figure 1*b*).

In an effort to better understand the population dynamic mechanisms responsible for the observed oscillations in density and the conditions under which these oscillations would occur, we developed a simple model of these population dynamics. In this model, *R* is the concentration of a limiting resource (μg ml^−1^), *B* is the density of the bacteria (cells per ml) and *T* is the concentration of the toxin (arbitrary units per ml). We assume a Monod (1949) model for resource concentration-dependent growth of the bacteria, *Ψ*(*R*)=*vR*/(*R*+*K*), where *v* is the maximum growth rate of the bacteria (per hour) and *K* is the concentration of the resource at which the bacteria are growing at half their maximum rate (μg ml^−1^). The limiting resource from a reservoir where it is maintained at a concentration of *C* (μg ml^−1^) enters a vessel of unit volume (1 ml) at a constant rate *w* (per hour), which is the same rate at which excess resources, bacteria and toxin are removed. The bacteria take up the resource at a rate proportional to their density, growth rate and a conversion efficiency parameter, *e* (μg) (Stewart & Levin 1973). In addition to being washed out with the flow, the toxin decays at a rate *d* (per hour).

The toxin kills the bacteria at a rate equal to the product of its concentration, the bacterial density and a constant *x*. We assume that the toxin is produced at a rate proportional to the product of its concentration, the density of bacteria and a constant *y*. Our biological justification for including the concentration of the toxin in its rate of production is the observation that other known secreted products in *S. pneumoniae*, such as the quorum-sensing peptide responsible for the induction of competence in *S. pneumoniae*, the competence-stimulating peptide (CSP) and the bacteriocin-like protein (BLP), involve a positive feedback (autocatalysis) between the concentration of these agents and their production (Havarstein *et al*. 1995, 1996; Pestova *et al*. 1996; de Saizieu et al. 2000; Berge *et al*. 2002).

With these definitions and assumptions, the rates of change in the density of bacteria and concentration of the resource and toxin are given by

(3.1) $$\frac{\text{d}R}{\text{d}t}=w(C-R)-\Psi (R)Be,$$

(3.2) $$\frac{\text{d}B}{\text{d}t}=\Psi (R)B-xBT-wB,$$

(3.3) $$\frac{\text{d}T}{\text{d}t}=yBT-\text{d}T-wT.$$

The density of bacteria that are killed by the toxin, *D*, is calculated from a fourth differential equation,

(3.4) $$\frac{\text{d}D}{\text{d}t}=xBT-wD.$$

**Figure 3:**
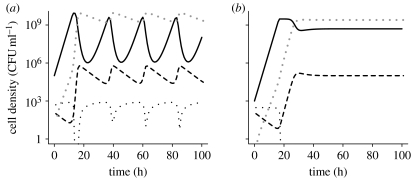
Simulation results: changes in the densities of bacteria *B* (black solid line); killed bacteria *D* (grey dotted line); and the concentration of the resource *R* (black dotted line) and toxin *T* (black dashed line). Parameters, (*a*) *C*=1000, *w*=0.1, *e*=10^−7^, *x*=5×10^−6^, *y*=4×10^−10^, *d*=0.10, *v*=1.0 and *k*=0.25 and (*b*) the same parameter values as in (*a*), except for the resource concentration in the reservoir, *C*=300.

To illustrate the properties of this model, we use numerical solutions to these differential equations. The growth rate parameter *v* used in these simulations is in the range estimated for *S. pneumoniae* R6 in the THY broth. The rate of flow, *w*, corresponded to that used in our experiments. The value of the Monod constant, *K*, is similar to that estimated for *Escherichia coli* in a glucose-limited minimal medium. For the maximum density (as determined by *C* and *e*), the toxin killing and production constants (*x* and *y*) and the toxin decay rate (*d*), we employed values that would produce the period and amplitude of the oscillations (figure 3*a*) similar to that observed in our experiments (figure 1*a*).

For an analytical understanding of the properties of this model and a dynamic summary of its properties, see the electronic supplementary material. The central point of this analysis is straightforward and interesting. Most importantly, this analysis shows that the resource concentration, *C*, and conversion efficiency, *e*, are not only the primary determinants of maximum population density but also control the dynamical behaviour of this system. To examine the transition between the different dynamic behaviours, we let *a*≡*ev*(*d*+*w*)/*wyC*, *b*≡*v*/*w* (note that necessarily *b*>1) and *f*≡(*d*+*w*)/*w* (with *k*≡*K*/*C*≪1, typically *k*∼10^−2^–10^−5^ holding for the different dynamics considered below). In accord with our analysis if and only if *b*>*a* (with the parameters used in figure 3, this corresponds to *C*>50), there exists a ‘three-species’ equilibrium with *R*, *B* and *T* all present. There is a very sharp transition in the dynamical behaviour when *a*≈1; and for *a*<1 (i.e. *C*>500), the oscillations generated decay very slowly (of the order of 10^5^ hours, with parameters and initial values of the variables employed in our simulations; figure 3*a*). For *b*>*a*>1, conditions that would be obtained when *C* is lower (300 μg ml^−1^), the oscillations are strongly damped (figure 3*b*).

**Figure 4:**
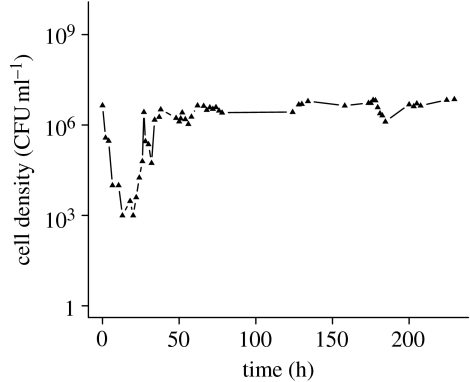
Changes in the density (CFU) of *S. pneumoniae* R6 grown in THY diluted by a factor of 28 in 0.85% saline. The dilution rate of this chemostat was approximately the same as that given in figure legend 1, approximately 0.1 per hour.

To test the validity of the model's prediction that the oscillations would be damped if the cell density is lower, we inoculated *S. pneumoniae* R6 into a chemostat with the THY broth diluted 28-fold. The results of this experiment are presented in figure 4. After the population recovered from an initial decline in density, which is attributable to an extended lag period in this nutrient-poor medium, the maximum density achieved is approximately two orders of magnitude lower than the maximum densities we observed in chemostats run with the full-strength THY broth (figure 1*a*). As predicted by the model, the oscillations in the diluted medium are considerably damped (figure 4) relative to that in the undiluted THY broth (figure 1*a*). While we cannot rule out the possibility that the physiological effects of low nutrient concentrations, rather than the reduction in cell density, are responsible for the dampening of the oscillations, we admit to some satisfaction with the consistency between our theoretical predictions and experimental results. After all, hypotheses can only be rejected.

To date, our efforts to characterize this toxin have been through exclusion experiments using strains of *S. pneumoniae R6, TIGR4* and *6A*, which do not produce known candidates for this toxin, and a limited biochemical characterization of the toxic supernatant.

One candidate for this toxin is H_2_O_2_, which is released by *S. pneumoniae* at concentrations that are lethal to the cells of this and other species of bacteria (Regev-Yochay *et al*. 2006, 2007). To test whether H_2_O_2_ is responsible for the oscillations, we added approximately 5000 units of catalase (Worthington Biochemical) to the 3 ml of soft agar used to grow the lawns of target cells, or incubated the supernatants from the chemostats for 30 min with 5000 units catalase before spotting. With both the treatments, the catalase would hydrolyse any H_2_O_2_. The results of this test indicated that H_2_O_2_ is not the toxin; lysis was observed on *S. pneumoniae* lawns containing catalase and with catalase-treated chemostat supernatants (figure 1*c*). Additional evidence that the toxin is not H_2_O_2_ comes from the observation of density oscillations in chemostats inoculated with *S. pneumoniae* with a deletion in the gene encoding the synthesis of pyruvate oxidase (TIGR4-Δ*spxB*) rendering this strain unable to produce hydrogen peroxide (Regev-Yochay *et al*. 2006, 2007; see figure S2 in the electronic supplementary material).

Our initial characterization of supernatants taken at the peak of toxin production suggests this agent is likely to be a protein. These supernatants do not generate zones of inhibition on lawns of *S. pneumoniae* R6 following incubation under denaturing conditions at relatively high temperatures or after protease treatment (figure 1*c*). Size fractionation using Microcon filters and the spot assay indicates that the relative size of the toxin is between 30 and 50 kDa (300 and 500 amino acids).

**Figure 5:**
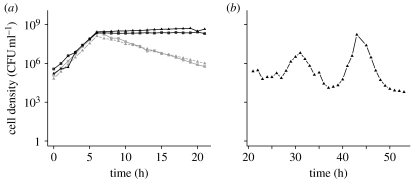
Changes in the density (CFU) of bacteria in batch and chemostat culture. (*a*) *S. pneumoniae* R6 *LytA*^+^ (grey squares and triangles) and *LytA*^−^ in THY batch culture (black squares and triangles). (*b*) A *LytA*^−^ strain of *S. pneumoniae* R6 in chemostat culture with a flow rate of 0.1 per ml in THY.

On first consideration, it may seem that the protein responsible for these oscillations is the same as that responsible for autolysis in batch cultures of *S. pneumoniae* (Sanchez-Puelles *et al*. 1986; Ronda *et al*. 1987; Havarstein *et al*. 2006). This does not appear to be the case. Mutants defective for the major pneumococcus lysin, *N*-acetylmuramoyl-l-alanine amidase (*lytA*), do not display autolysis in batch culture (figure 5*a*), yet they oscillate in density when introduced into a chemostat (figure 5*b*).

It is somewhat gratifying that the products of *lytA* and hydrogen peroxide can be ruled out as the toxins, because there is no evidence for their being produced autocatalytically as our model predicts they would have to be. On the other side, our experiments have also ruled out ‘suspects’ that directly or indirectly induce lysis in pneumococcus and for which there is evidence for autocatalytic production: CSP and BLP (Claverys & Havarstein 2002; Peterson *et al*. 2004; Dawid *et al*. 2007; Lux *et al*. 2007). In chemostat culture, mutants that are defective for the production of these secreted peptides (plus the CSP receptor), *comC, comD* and *blpR*, oscillate in a manner similar to wild-type strain R6 in chemostat culture (see figures S3 and S4 in the electronic supplementary material). In addition, a mutant defective in the competence response regulator (Δ-*ComE*), which is unable to induce competence or activate downstream genes regulated by competence induction, also shows oscillatory dynamics in a chemostat similar to the parental strains from which it was derived (see figure S5 in the electronic supplementary material).

A particularly appealing candidate for the toxin responsible for the density oscillations is the murine hydrolase coded for by the gene *CbpD*. As Johnsborg *et al*. (2008) recently demonstrated using a β-galactosidase release assay, murein hydrolase lyses pneumococcus in liquid culture. This enzyme is approximately the molecular weight we estimated for the toxin in the supernatants of our chemostats, and, as we show below, the host range of bacteria sensitive to the *S. pneumoniae* R6 murine hydrolase resembles that of the supernatant from the oscillating *S. pneumoniae* R6 chemostats (Johnsborg *et al*. 2008). But unfortunately, strains of pneumococcus R6 knocked out for the production of murein hydrolase protein, Δ*CbpD*, oscillate to an extent no different from the *CbpD+* strain from whence it was derived (see figure S5 in the electronic supplementary material), and the supernatant of these Δ*CbpD* chemostats produced zones of inhibition on R6 lawns similar to those we observed with *CbpD*+ strains (data not shown).

Using the spot assay we tested the cross-sensitivity of R6 and the three clinical isolates to their respective secreted toxins. For each supernatant, zones of inhibition were observed on lawns of all four strains (figure 2*b*). To further explore the host range of the R6 toxin, we tested for lysis on lawns of the other *Streptococcus* species. The results of these assays suggest that closely, but not distantly, related species of *Streptococcus* are susceptible to the *S. pneumoniae* R6 toxin (see figure S7 in the electronic supplementary material).

The observations that the activity of the toxin driving these oscillations does not appear to be strain specific and lyses other, closely related, species of *Streptococcus* raise the possibility (hypothesis) that the ecological role of these toxins is allelopathy. In this interpretation, these toxins either prevent the invasion of established populations by competing strains and species sensitive to this toxin or facilitate invasion into established populations of toxin-sensitive strain species. At this time, we have not tested this allelopathy hypothesis experimentally. However, using an extension of the model equations (3.1)–(3.3), we have explored the *a priori* plausibility of this hypothesis by ascertaining the conditions under which self-killing toxins would prevent invasion of competitors into established populations of toxin-producing bacteria. In this extended version of our model, there is a second population of bacteria, *B*_2_, that is susceptible to the toxin produced by the first, *B*_1_, but does not produce that toxin. The system of equations describing this situation is

(3.5) $$\frac{\text{d}R}{\text{d}t}=w(C-R)-\Psi (R)e(V_{1}B_{1}+V_{2}B_{2}),$$

(3.6) $$\frac{\text{d}B_{1}}{\text{d}t}=\Psi (R)V_{1}B_{1}-x_{1}B_{1}T-wB_{1},$$

(3.7) $$\frac{\text{d}B_{2}}{\text{d}t}=\Psi (R)V_{2}B_{2}-x_{2}B_{2}T-wB_{2},$$

(3.8) $$\frac{\text{d}T}{\text{d}t}=yB_{1}T-d_{\text{T}}T-wT,$$

where *Ψ*(*R*)=*vR*/(*R*+*K*); *V*_1_ and *V*_2_ are the maximum growth rates of these bacteria; and *x*_1_ and *x*_2_ are the killing rates of the producing and invading bacteria, respectively. For convenience, we assume that the efficiency of conversion of resource into bacteria biomass, *e*, and the Monod constant, *k*, are identical for *B*_1_ and *B*_2_. As in the previous model, *y* is the rate constant of production of the toxin and *d*_T_ is the inverse of the half-life of the toxin.

As indicated in the analysis of the properties of this model in the electronic supplementary material, there are conditions under which the production of a toxin that is lethal to the bacteria that produce it can prevent invasion by a toxin-sensitive population, even when that invader has a higher growth rate than the established population *V*_2_>*V*_1_. This, however, requires that the ratio of the rates of toxin-mediated killing of the invading and established strain, *x*_2_/*x*_1_, is larger than *V*_2_/*V*_1_ and that the density of *B*_1_ is sufficiently high to produce adequate concentrations of the toxin.

**Figure 6:**
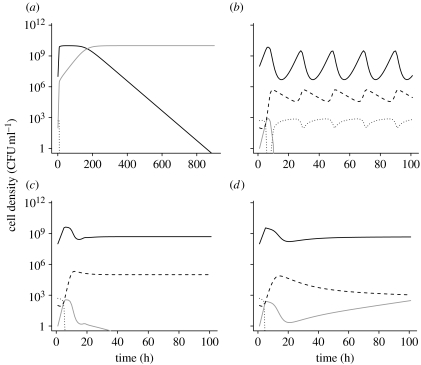
Simulation results: invasion of a clone with higher intrinsic fitness. Changes in the densities of the bacterial populations and concentrations of the toxin (black dashed line) and resource (black dotted line). The parameter values for this simulation are: *V*_1_=1.0; *V*_2_=1.5; *k*=0.25; *e*=1×10^−7^; *y*=4×10^−10^; *ω*=0.1; *x*_1_=5×10^−6^; *x*_2_=9×10^−6^; and *d*=0.1. In these simulations, a single cell of B_2_ (grey solid line) is introduced into the populations of *B*_1_ (black solid line) at an initial density of 10^8^. In (*b*)–(*d*), the initial concentration of the toxin is 50 units per ml. (*a*) Invasion in the absence of toxin production (*y*=0). (*b*) Failure to invade a high-density (*C*=1000) oscillating toxin-producing population. (*c*) Failure to invade a lower density (*C*=300) non-oscillating toxin-producing population. (*d*) Invasion in the absence of the maintenance of toxin production because of very low resource concentration (*C*=50). In these simulations, when the density of the invading *B*_2_<0.5, the simulation *B*_2_ was set equal to zero.

As can be seen in the simulations presented in figure 6*a*, in the absence of the toxin, the higher fitness clone, *B*_2_, will invade and replace the established population *B*_1_. If, however, the average density of *B*_1_ is high and *x*_2_/*x*_1_>*V*_2_/*V*_1_, the invasion of *B*_2_ can be prevented. This is obtained when the density of *B*_1_ oscillates (figure 6*b*) and when the resource concentration is too low for this established population to oscillate (figure 6*c*). However, if the density of *B*_1_ and thus the concentration of toxin in the environment are too low, the clone with the greater growth rate will invade (figure 6*d*), albeit at a rate lower than in the absence of toxin production (figure 6*a*). It should be noted, however, that the conditions for *B*_2_ to invade an established population of *B*_1_ are to some extent dependent on the initial density of *B*_1_ and thus the concentration of the toxin, T, in the environment. If the density of *B*_1_ is initially low, *B*_2_ can invade under conditions where it would not invade a toxin-producing *B*_1_ population with an initially higher density (simulation results not shown). Our numerical simulation results indicate with reasonable parameter conditions, that even if *B*_2_ has a higher growth rate fitness than *B*_1_ and produces a toxin that kills *B*_1_, when rare *B*_2_ cannot colonize a community dominated by this high-density, toxin-producing established strain (results not shown).

## 4. Discussion

When we set out to use chemostat cultures to estimate the rate of transformation-mediated chromosomal gene recombination in *S. pneumoniae* (now a tale for another report), we did not anticipate the profound oscillations in density reported here; this study is founded on a serendipitous observation. From a population dynamic perspective, these oscillations can be accounted for by the autocatalytic production of an agent, a toxin, which either directly lyses or induces autolysis in other cells of the same clone. The results of our experiments are consistent with the predictions of this model. The concentration of this toxin appears to reach its maximum shortly after the density of the oscillating population reaches its apex and these oscillations are damped when the density of the culture is low.

We have yet to characterize the toxin responsible for these oscillations or determine the physiological and molecular mechanisms by which it acts. Our results, however, indicate that this toxin is likely to be a protein of between 30 and 50 kDa. By exclusion, our experiments suggest that this toxin is novel in the sense of not being described or characterized earlier. Using strains of *S. pneumoniae* deleted for the genes coding for candidates for this toxin, we have excluded the following potential toxins or genetic systems involved in its production:

- *N*-acetylmuramoyl-l-alanine amidase, *lytA*, which is responsible for autolysis in batch culture,
- the competence-inducing peptide, *comC*,
- the BLP system *blpR*,
- murein hydrolase which lyses pneumococcus in liquid culture, *CdpB*, and
- the cognate response regulator for the competence system, *comE*.

The densities of strains that do not produce these peptides and proteins oscillate in a manner similar to that observed in the parental strains from which they were constructed. Moreover, using catalase and a strain that does not produce hydrogen peroxide, we have also excluded this oxidizing agent as being responsible for these density oscillations.

Although density oscillations were observed for all the strains we examined, there appears to be strain differences in the densities reached in these chemostats, with the same media and flow rates, and in the amplitude and, possibly, the period of these oscillations. For two reasons, we have not explored the variation in these dynamics systematically. First, strain variation in these dynamics is secondary to the main focus of this investigation, which is the population dynamic processes responsible for these oscillations and the ecological/evolutionary reason bacteria produce the self-killing toxins generating these oscillations. Second, the frequent sampling of viable cell density (CFU data) required for a quantitatively accurate characterization of oscillations with periods of 30 hours or so is an onerous task that we cannot justify doing given the motivation for this study.

Probably, for many readers and certainly for the authors, the most intriguing questions raised by this study are the ecological role and thereby the selection pressure responsible for the evolution of this oscillation-driving toxin. Despite the fact that this phenomenon occurs with natural isolates as well as laboratory strains of *S. pneumoniae*, it is conceivable that density oscillations of the amplitude observed in figure 1*a* are an artefact of laboratory culture. In their natural habitat within the human nasopharynx, *S. pneumoniae* may never reach the densities where these oscillations occur. As predicted by our model (figure 3*b*) and demonstrated experimentally (figure 4), oscillations in density of the amplitude observed in figure 1 are not anticipated in environments that can only support low-density populations. However, whether these oscillations are an artefact of culture conditions or not still remains necessary to account for why, evolutionarily, *S. pneumoniae* produce this and the other toxins that kill genetically identical bacteria.

If the killing of genetically identical members of the same population were the sole function of the toxin, the capacity to produce it or respond to its action would not be favoured by natural selection. If, however, the agent responsible for this killing provides a survival or growth advantage to the producing clone in its natural habitat, its production could be favoured by selection even if some or even many cells of the producing clone are killed. In §1, we briefly described three hypotheses that have been proposed to account for how the production of these suicidal agents could provide an advantage to the toxin-producing population:

- Cannibalism: to acquire resources (nutrients and carbon sources) from the killed cells that would increase the likelihood of survival of the whole clone in times of dearth (Sanchez-Puelles *et al*. 1986; Ronda *et al*. 1987; Lewis 2000; Claverys & Havarstein 2007) or postpone entry into a non-replicating state, sporulation (Gonzalez-Pastor *et al*. 2003).
- The acquisition of adaptive genes through transformation of DNA by lysing cells of other clones and species that bear these genes (Lewis 2000; Steinmoen *et al*. 2002, 2003; Claverys & Havarstein 2007).
- The release of other agents, such as virulence factors that would enable the population to colonize a new habitat or, for parasitic bacteria, an uncolonized host (Pinas *et al*. 2008).

Here, we propose a fourth hypothesis for the production of these clone suicidal toxins: allelopathy to prevent invasion of established populations. We find this hypothesis appealing not only because we presented it, but also because the toxin responsible for the observed oscillations kills other clones of the same species as well as closely related and thereby potentially competing species. Our theoretical analyses suggest that there are relatively broad conditions under which the production of toxins that kill bacteria of the same genotype that produce them can prevent invasion of established populations by toxin-sensitive populations that would otherwise invade because of a growth rate advantage. The necessary conditions for this to occur are that the invader is more sensitive to killing by the toxin than the resident and the concentration of the toxin in the habitat is sufficiently great for toxin-mediated killing to override the growth rate advantage of the invading population. A testable prediction of this hypothesis is that strains would be less sensitive to killing by the toxins they produce than they are to the toxins produced by other strains and species.

In this paper, we have not formally explored the other face of this allelopathy hypothesis, i.e. to facilitate the invasion of established populations of bacteria. For two reasons, we would anticipate that conditions for the invasion of a clone producing a clonal suicide toxin in mass (liquid) culture would be highly restrictive (Chao & Levin 1981; Levin 1988). (i) Unlike bacteriocins, there is no evidence for immunity to this toxin by the producing clone. (ii) All of the strains and species of *Streptococcus* we have examined that are sensitive to this toxin also produce that toxin or a variant of it to which all the other strains and species are sensitive. To be sure, in physically structured habitats, where bacteria are maintained as colonies rather than planktonic cells, there are broad conditions under which bacteriocin production can facilitate invasion of established sensitive populations (Wiener 1996; Kerr *et al*. 2002). Whether this would also be the case for clonal suicide toxins, and how the production of these toxins would affect the population dynamics of bacteria in physically structured habitats, remains to be seen and are intriguing problems for another time.

The four hypotheses for the evolution and maintenance of self-killing toxins considered here are not mutually exclusive and, to our knowledge, none of them has been adequately tested, much less unambiguously supported experimentally. To be sure, there is good evidence that the rate of transformation-mediated recombination is greater for *S. pneumoniae* that produce a lysis-inducing murein hydrolase protein than for otherwise isogenic cells that do not (Johnsborg *et al*. 2008). On the other hand, we know of no evidence that this higher rate of recombination would provide a sufficient advantage to the murein hydrolase-producing population to overcome likely fitness costs associated with synthesizing this protein and its killing isogenic cells.

Particularly intriguing from an evolutionary perspective is why pneumococcus remains susceptible to the toxins it produces. Even if the densities within natural habitats are too low for the toxin considered here to generate oscillations of the sort that motivated this study, if it is produced at all some cells would still be killed by its action. In other words, there would be continuous and, possibly, intense selection for resistance to this toxin. Nevertheless, resistance to this toxin has not been observed in *S. pneumoniae* chemostats maintained for three and a half months (D.E.R and O.E.C, unpublished data). Perhaps more compellingly, since clinical (wild) isolates of *S. pneumoniae* also oscillated in chemostats and produce the toxin, it is reasonable to assume that resistance to the toxin has not evolved even when there was plenty of time for this evolution to occur. Could it be that viable and fit mutants resistant to the toxin cannot be generated by mutation or acquired by horizontal gene transfer?

Although a largely commensal bacteria that asymptomatically colonizes many humans, *S. pneumoniae* is also responsible for a number of invasive infections, e.g. otitis media, bacteremias, meningitis and pneumonia. While existing antibiotics have been successful in treating invasive pneumococcus disease and resistance infrequently prevents effective chemotherapy (Yu *et al*. 2003), this may not be true in the future. Anti-pneumococcus drugs with novel targets will be needed. Could the toxin responsible for the lysis reported here be developed into an effective and safe drug to treat pneumococcus infections? This would be particularly appealing if indeed viable strains of pneumococcus resistant to this toxin cannot be generated.

## Figures and Tables

**Table 1:** List of strains and *streptococcus* species used in this investigation. (The source of each strain and the relevant genotypic features for this study are provided. Genetic marker employed (when relevant) is also provided.)

strain/species ID	relevant genotypic features	genetic marker	source
R6	non-encapsulated derivative of D39		laboratory isolate
*LytA*	Δ-*LytA* in the R6 background		A. Tomasz
Δ-*ComC* originally FP5	Δ-*ComC* in the RX1 background	chloramphenicol resistant	Iannelli *et al*. (2005)
Δ-*ComD* originally FP48	Δ-*ComD* in the RX1 background	kanamycin resistant	Iannelli *et al*. (2005)
PMEN-6	PMEN-6 multiply antibiotic resistant clinical isolate, serotype 23F		McGee
PMEN-18	PMEN-18 multiply antibiotic resistant clinical isolate, serotype 14		L. McGee
PMEN-20	PMEN-20 multiply antibiotic resistant clinical isolate, serotype 6B		L. McGee
TIGR4	encapsulated strain	streptomycin resistant	laboratory strain (Regev-Yochay *et al*. 2007)
TIGR4 Δ-*spxB*	Δ-*spxB* in the TIGR4 background	kanamycin resistant	Regev-Yochay *et al*. (2007)
6A	encapsulated strain of serotype 6A		Dawid *et al*. (2007)
Δ-*blpR*	Δ-*blpR* in the 6A background	erythromycin resistant	Dawid *et al*. (2007)
RH-1	R6 derivative, but *ebg*::*spc*	Ery^R^, Spc^R^,	Johnsborg *et al*. (2008)
RH-3	RH1 but Δ-*ComE*	Ery^R^, Spc^R^, Cm^R^, Kan^R^	Johnsborg *et al*. (2008)
RH-17	RH1 but Δ-*Cbdp*	Ery^R^, Spc^R^, Kan^R^	Johnsborg *et al*. (2008)
*Streptococcus mitis*			ATCC no. 49456
*Streptococcus oralis*			ATCC no. 35037
*Streptococcus sanguinis*			ATCC no. 10556
*Streptococcus salivarius*			ATCC no. 7073
*Streptococcus gordonii*			ATCC no. 10558
*Streptococcus pyogenes*			ATCC no. 21060
*Streptococcus australis*			ATCC no. 700641

## Supplementary Material

Additional online material Oscillations in continuous culture populations of *Streptococcus*: population dynamics and the evolution of clonal suicide
