# Supplementary material for: Oscillations in continuous culture populations of Streptococcus pneumoniae: population dynamics and the evolution of clonal suicide
Source: Proc Biol Sci. 2008 Dec 3;276(1659):999–1008. doi: 10.1098/rspb.2008.1415 (PMC2679064; doi:10.1098/rspb.2008.1415)
Supplement: Additional online material — Oscillations in continuous culture populations of Streptococcus: population dynamics and the evolution of clonal suicide [file rspb20081415s01.pdf]

Supplementary Online Material: Oscillations in Continuous Culture Populations of *Streptococcus pneumoniae*: Population Dynamics and the Evolution of Clonal Suicide

Omar E. Cornejo[1], Daniel E. Rozen[2], Robert M. May[3], Bruce R. Levin[1*]

[1] Department of Biology, Emory University, 1510 Clifton Road, Atlanta, GA 30322, USA
[2] Faculty of Life Sciences, University of Manchester, Manchester, M13 9PT, UK
[3] Department of Zoology, University of Oxford, Oxford, OX1 3PS, UK
* Corresponding author,

December 3, 2008

Contents

1 Mathematical Model. Analysis 2

2 Strain deficient in the production of Hydrogen peroxide oscillates 7

3 Strains deficient in the induction of competence (*comC* and *ComD* deficient) and bacteriocin like protein (*blpR* deficient) systems oscillate 7

4 Strains deficient in the production of murein hydrolase, *CbpD*, oscillate 8

5 Assisted suicide protects established populations for invasion of higher fitness clones or species. Model and analysis. 10

6 Toxin sensitivity of other species of *Streptococcus* 13

1 Mathematical Model. Analysis

The simple version of the model is described by the following set of equations

$$\begin{aligned} \dot{R} &= \omega(C - R) - \Psi(R)eB && (1) \\ \dot{B} &= \Psi(R)B - \omega B - xBT && (2) \\ \dot{T} &= yBT - \omega T - dT && (3) \end{aligned}$$

where, $\Psi(R) = \nu R/(K + R)$

These equations can be rewritten in dimensionless form by rescaling the resource (R), bacterium (B), and toxin (T) variables as:

$$R' = R/C, \quad B' = yB/f\omega, \quad T' = xT/\omega \qquad (4)$$

This gives:

$$\begin{aligned} \dot{R}' &= 1 - R' - aB'\psi(R') && (5) \\ \dot{B}' &= B'(b\psi(R') - 1 - T) && (6) \\ \dot{T}' &= fT'(B' - 1) && (7) \end{aligned}$$

with $\psi(R')$ defined as $\psi(R') = R'/(R' + \kappa)$

Here we have also rescaled the time variable as $t' = \omega t$. The dimensionless combinations of parameters which determine the system's dynamical behavior are:

$$f = (\omega + d)/\omega, \quad \kappa = K/C, \quad a = ef\nu/yC, \quad b = \nu/\omega. \qquad (8)$$

The possible stationary points (equilibrium points*) of this system, R^*, B^*, and T^*, are found by putting $\dot{R} = \dot{B} = \dot{T} = 0$, and solving the ensuing algebraic equations. There are three such stationary points.

From this point we dropped the superscript prime (′) on each variable. $B' \to B$, $R' \to R$, $T' \to T$.

The first –"resource only"– such point has:

$$B^* = T^* = 0, \quad \text{and} \quad R^* = 1 \tag{9}$$

A linearized stability analysis shows this point is stable if, and only if,

$$\kappa/(b-1) > 1 \tag{10}$$

Failing this, an arbitrary small introduction of bacterium will grow ($\dot{B} > 0$).

The second –"plant-hervibore"– point has

$$R^* = \kappa/(b-1), \quad B^* = (1-R^*)b/a, \quad T^* = 0 \tag{11}$$

This equilibrium is stable if, and only if,

$$(a/b) + \kappa/(b-1) > 1 > \kappa/(b-1) \tag{12}$$

Within this domain, any introduction of T decays to zero, and the system returns to the R^*, B^* equilibrium with damped oscillations if $\kappa > (b-1)^2/(2b-1)$, or via exponential damping otherwise.

Third, and the most interesting, is a "3 species" (a "plant-hervibore-predator") equilibrium solution with

$$B^* = 1, \quad R^* = -D + (D^2 + \kappa)^{1/2}, \quad T^* = (b/a)(1-R^*) - 1. \tag{13}$$

Here, D is defined for notational convenience as $D = (a + \kappa - 1)/2$. This equilibrium solution is stable with all the three components persisting together, if and only if

$$1 > (a/b) + \kappa/(b-1) \tag{14}$$

For the experimental system, representative values of the parameters are $\omega = 0.1$ ml $hour^{-1}$, $x = 5 \times 10^{-6}$ ml $cells^{-1}$ $a.u.t.^{-1}$, $e = 10^{-7}$ μg, $y = 4 \times 10^{-10}$ ml $cells^{-1}$ $a.u.t.^{-1}$, $K = 0.25$ μg ml^{-1}, $\nu = 1$ $cells$ $hour^{-1}$, $d = 0.1$ $hour^{-1}$ (the $a.u.t$ are defined as **"arbitrary units of toxin"**). The parameter characterizing the **resource/chemostat concentration**, C, take various values, but always $C >> K$. Given that C is the control variable and κ represents it, the definition of a can be changed to $a = \alpha\kappa$ with α defined below. For the dimensionless parameters of Eq.(8) we thus have $f = 2$, $b = 10$, $a = \alpha\kappa$ (with $\alpha = 2000$), and $\kappa = 0.25/C$, with C having values from a few tens to a few thousands. That is, $\kappa << 1$, whereas a can be less or greater than 1, and the defintion of α is as follows:

$$\alpha = \frac{ef\nu}{yK}, \tag{15}$$

and it is important to remember that according to our definitions $K = \kappa C$.

The dynamics of this 3-species system (represented in Figure S1), as κ varies (corresponding to the chemostat resource concentration C varying), are interesting, with a rather sharp transition in dynamical behavior at the point where $a = 1 - \kappa \approx 1$. For the illustrative parameters above, this corresponds to $C = 500$. For a smaller than this ($a < 1$), and remembering $\kappa \ll 1$, for all C-values of interest, we have $D = (a + \kappa - 1)/2 < 0$, resulting in $R^* \approx 1 - a$, $B^* = 1$, $T \approx (b - 1)$. In this region the system approaches its stable equilibrium point via very weakly damped oscillations. The oscillations have a period, P, approximatelly given by

$$P \approx 2\pi[f(b-1)]^{-1/2}/\omega \tag{16}$$

whilst the damping time, τ, is very long of the order

$$\tau \sim 2[1 + f(b-1)][1-a]^2/[ab\omega\kappa] \tag{17}$$

Here, these expressions are given in absolute time (not rescaled), hence the factor ω.

The dynamical behavior changes abruptly when κ —- although still much less than 1 —- approaches the value such that $a = \alpha\kappa$ (with $\alpha \gg 1$) is essentially one: $a = 1+$ (a few multiples of)κ. In this tiny window, D $\sim K$ and $R^* \approx \kappa^{1/2}$, while still $B^* = 1$ and $T^* = b - 1$. The system still approaches equilibrium via damped oscillations, but now with the period (P) and the damping time (τ) are of comparable magnitude: P is much as above, but now $\tau \sim [4 + f(b-1)]/b\omega$.

Finally, for $a > 1$ (by a few multiples of κ), we move into a regime where $R^* \approx \kappa/(a-1) << 1$, $B^* = 1$, $T^* = (b-a)/a$.

Perturbations to this equilibrium point decay back to it; the characteristic damping time is $\tau \sim 1/[(\beta - \gamma)\omega]$, with the definitions $\beta = b/2a$ and $\gamma = (\beta^2 - 2f\beta + f)^{1/2}$.

As a increases, β and thence γ decrease, until we have $\gamma = 0$ when $\beta = f - (f^2 - f)^{1/2}$. Beyond this point, the system returns to its equilibrium point via damped oscillations, whose period ($T \sim 1/ \mid \gamma \mid \omega$) and damping time ($\tau \sim 1/\beta$) have comparable magnitudes. Eventually, once a becomes sufficiently large that $a > b[1 - \kappa/(b-1)]$, the 3-species equilibrium is no longer stable, and the toxin T can no longer be maintained in the system.

Figure **??** illustrates this range of behavior, and in particular the very abrupt transition around the point $a = 1$ (which for the illustrative parameter values outlined above, corresponds to $C = 500$): for C-values above this, there are very weakly damped oscillations; below it, there is exponential damping if $341 < C < 500$, and damped oscillations for $50 < C < 341$. Once $a > b[1 - \kappa/(b-1)]$, corresponding here to $C < 50$, the toxin can no longer be maintained, and we have a stable "resource - bacterium" system R^*-B^*. For implausibly small C-values ($C < 1/36$), the only stable state is the "resource-only" system, with neither bacterium nor toxin. Again, and at the risk of being repetitive, the composite parameteres a, b, and f allows us to examine the transition between the different dynamic behaviours that the system might experience as the result of changes in the resource concentration C.

It should be emphasized that the stability analysis presented above is, strictly speaking, valid only for small perturbations (i.e. a linearized analysis). But – although we have not found a Lyapunov function for the above system – previous experience suggests the global dynamical properties march with the local ones. Extensive numerical simulations support this suggestion.

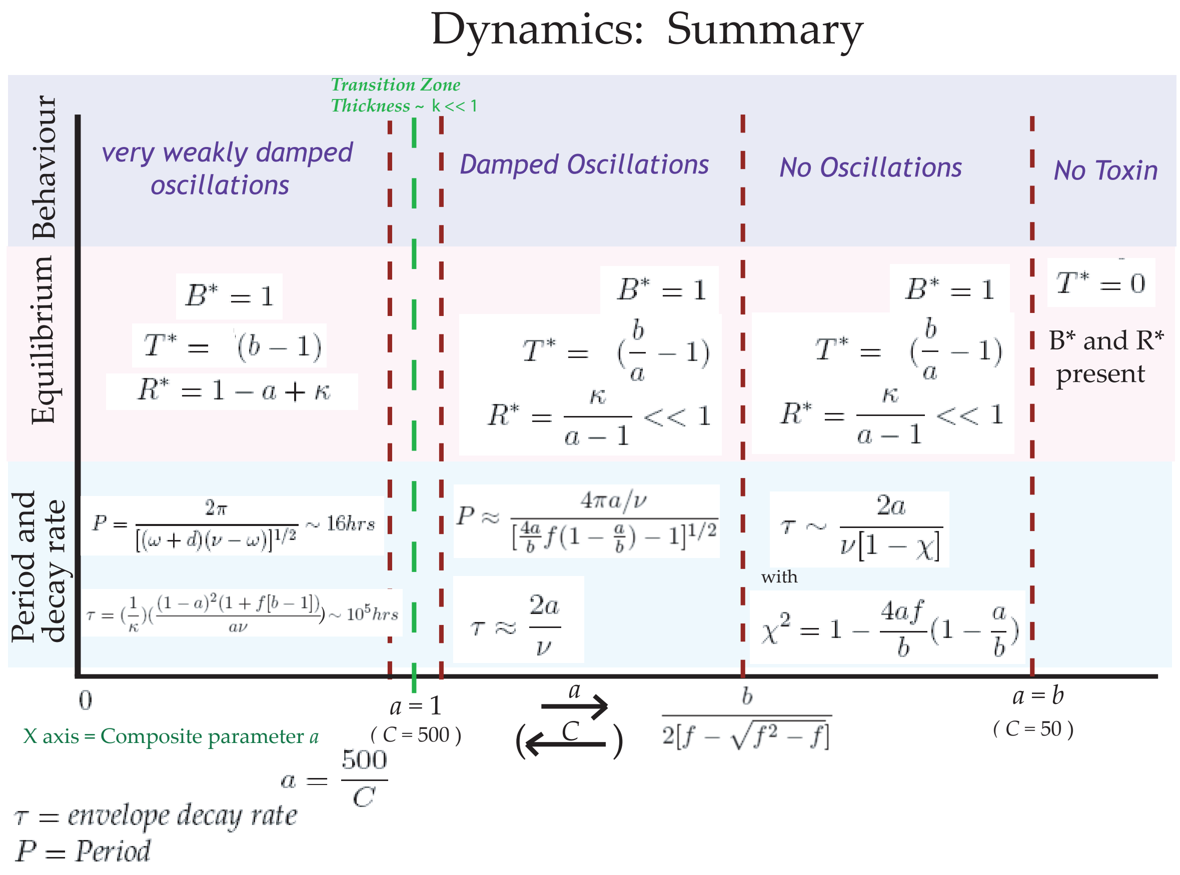

Figure S1: Diagram of the Dynamics predicted by the model. The space of possible dynamics is shown as a function of values the composite paramter a. It is shown in different boxes (from the top to the bottom) the Predicted Dynamics, the predicted equilibrium state for each range of a, The characteristic oscillation period (P) and envelope decay rate (τ) of the oscillations for the differen behaviours

2 Strain deficient in the production of Hydrogen peroxide oscillates

Pneumococcus inhibits the replication or lyses *Staphylococcus aureus* by the release of H_2O_2 (Regev-Yochay et al. 2007). This raises the possibility that hydrogen peroxide could be the toxin responsible for the observed oscillations of *S. pneumoniae* R6 chemostat culture. The results of experiments with catalase reported in our article are inconsistent with this interpretation. Also inconsistent are the experiments we performed with a strain deficient in the production of pyruvate oxidase, an exzyme required for the production of hydrogen peroxide. In chemostat culture this strain oscillates, Figure S2.

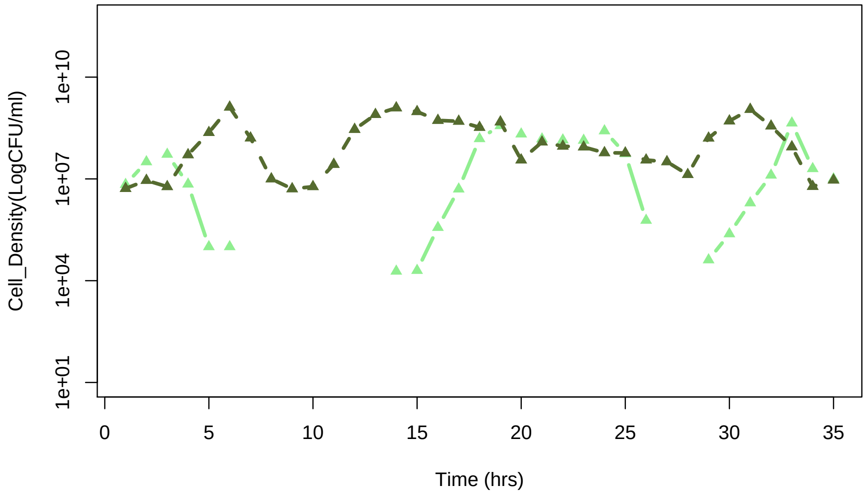

Figure S2: Changes in density (CFU) of *S. pneumoniae* strain (TIGR4 background) deficient for the production of pyruvate oxidase (Δ-*spxB*) an enzyme required for the production of H_2O_2 (dark green lines and triangles) and the *spxB*+ control (light green lines and spots).

3 Strains deficient in the induction of competence (*comC* and *ComD* deficient) and bacteriocin like protein (*blpR* deficient) systems oscillate

The competence peptide of *S. pneumoniae*, which appears to be produced in an autocatalytic manner similar to toxin postulated here, is a reasonable candidate for the agent (toxin or regulatory factor involved in the production of the toxin) driving the oscillations.

To test this hypothesis, we inoculated chemostats with mutants defective in the production of the competence peptide (Δ-*ComC*) or in the receptor of the competence peptide (Δ-*ComD*). These strains oscillate (Figure S3).

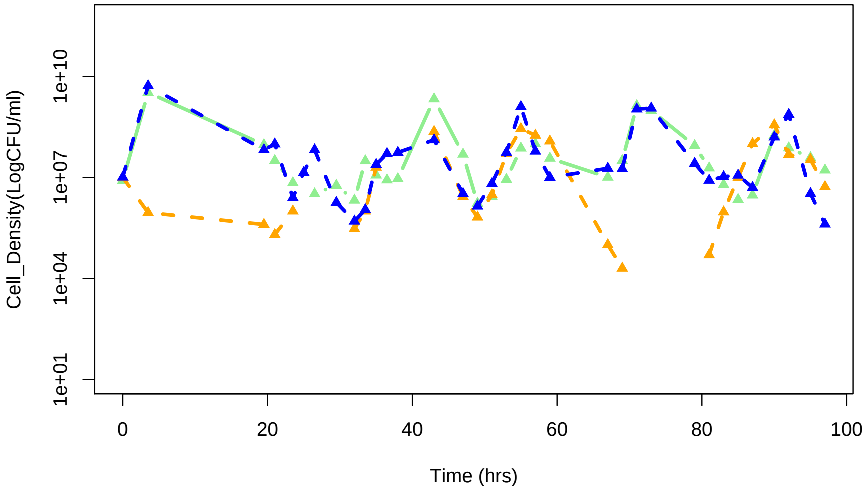

Figure S3: Changes in density in chemostat culture of *S. pneumoniae* wild type R6 (dark blue) and R6 deficient in the production of the competence peptide Δ-*ComC* (light green), and its receptor Δ-*ComD*) in light orange.

A similar behaviour is found in strains in which the regulatory gene of the *blp* system, also known to operate autocatalitically, has been knocked out. That is, it shows oscillatory dynamics similar to the parental strain (Figure S4)

4 Strains deficient in the production of murein hydrolase, *CbpD*, oscillate

As estated in the previous section the competence peptide of *S. pneumoniae*, which appears to be produced in an autocatalytic manner, does not oscillate. Additional evidence is found in a strain that lacks the production o the general response regulator *ComE* of the competence system (see Figure S5). Moreover, the lysing protein murein hydrolase, produced by *CbpD*, and seemingly regulated by the competence system, does not seem to prevent the strains from oscillating (see Figure S5), and we consider it can be ruled out as a potential oscillation driving agent in this system.

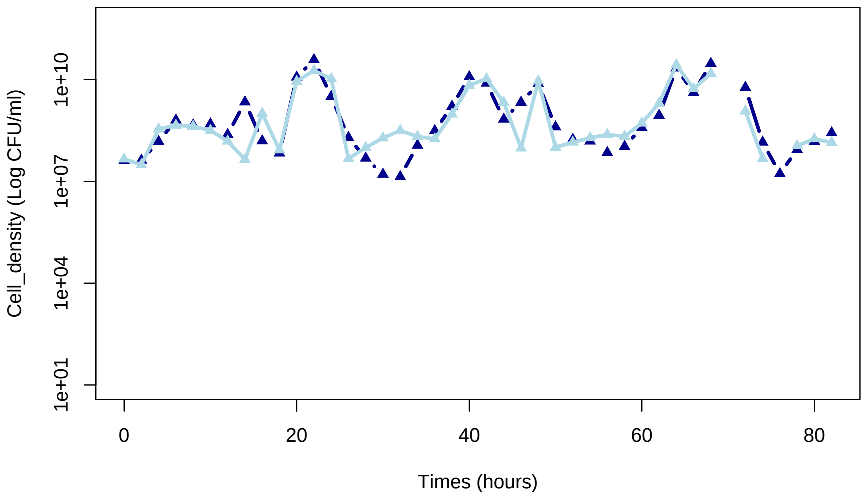

Figure S4: Chemostat dynamics of the *S. pneumoniae* 6A strain (serotype 23) deficient in the production of the regulatory peptide of the *blp* regulon (Δ-*blpR* Light Blue). The wild type is shown in Dark Blue.

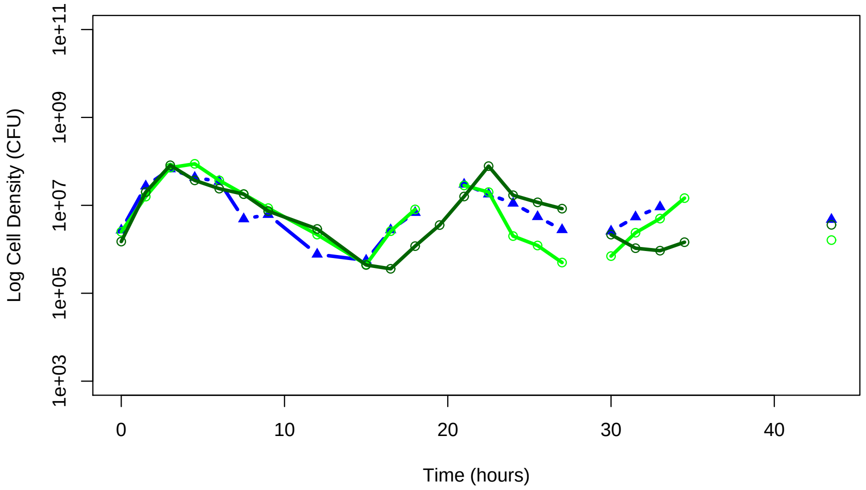

Figure S5: Changes in density in chemostat culture: *S. pneumoniae* RH1 wild type (blue). RH17 a derivative of RH1 deficient in the production of the murein hydrolase protein, Δ-*CbpD*, (dark green), RH3 a derivative of RH1 deficient in the production of the cognate response regulator of the competence system, Δ-*ComE*, (green) (Johnsborg et al. 2008)

5 Assisted suicide protects established populations for invasion of higher fitness clones or species. Model and analysis.

In our report, we postulate that despite the killing of members of the same clone, assisted suicide could have evolved and be maintained as a mechanism to prevent established populations from invasion by bacteria of different clones or species with greater intrinsic fitness. The necessary condition for this is the invading clone is sufficiently sensitive to the toxin at its concentration in an established population of *Streptococcus pneumoniae* to have a net disadvantage relative to members of the established clone.

To begin exploring these invasion prevention conditions quantitative way, we expanded our model (equations 1-3) to include a second population of bacteria, B_2 and add a subscript 1 to the variable designating the toxin-producing species, B_1. We assume both species are limited by the same resource which they consume in a Monod-like fashion with for simplicity the same values of k and e. These two populations can have different maximum growth rates, V_1 and V_2, and different sensitivities to the toxin. With these definitions and assumptions, the rates of change in the densities of the different populations are given by,

$$\dot{R} = (C - R)\omega - e\Psi(R)(B_1V_1 + B_2V_2) \quad (18)$$
$$\dot{B_1} = \Psi(R)V_1B_1 - \omega B_1 - x_1B_1T \quad (19)$$
$$\dot{B_2} = \Psi(R)V_2B_2 - \omega B_2 - x_2B_2T \quad (20)$$
$$\dot{T} = yB_1T - \omega T - dT \quad (21)$$

where, $\Psi(R) = \nu R/(K + R)$ as in the previous model. V_1 and V_2 are the maximum growth rates of these bacteria, and x_1 and x_2 are the killing rates of the producing and invading bacteria respectively. For convenience we assume that the efficiency of conversion of resource into bacteria biomass, e, and the Monod constant, k, are identical for B_1 and B_2. As in the previous model y is the rate constant of production of the toxin and d is the inverse of the half-life of the toxin.

For analytical tractability in the above model we are assuming that B_2 does not produce the toxin. This assumption can be justified by our concern with the conditions for invasion of established populations of the toxin-producing strain B_1 by B_2 when it is rare. Even if the per-capita rate of production of the B_2 toxin was great and the established population

highly sensitive to it, as long as the density of the invading population is low, the concentration of the toxin produced by it would be too low to cause a perceptible effect on the much more numerous established population.

Suppose B_2 tries to invade the $R - B_1 - T$ system. That is, for entry/growth of a tiny amount of B_2, we have:

$$\dot{B}_2 = B_2(\psi(R^*)\nu_2 - \omega - x_2 T^*) \tag{22}$$

where, R^*, T^* are given by the (equilibrium) solutions of the $R - B_1 - T$ system being invaded.
Clearly, invasion is possible if, and only if,

$$\nu_2 \psi^* > \omega + x_2 T^* \tag{23}$$

But ψ^* is given by $\nu_1 \psi^* = \omega + x_1 T$, so Eq.(23) becomes

$$\frac{\nu_2}{\nu_1}(\omega + x_1 T) > \omega + x_2 T \tag{24}$$

i.e

$$\left(\frac{\nu_2 - \nu_1}{\nu_1}\right) > \left(x_2 - \frac{\nu_2 x_1}{\nu_1}\right) \frac{T^*}{\omega} \tag{25}$$

with T^* from our earlier analysis of $R - B_1 - T$ system.

Clearly, if $\nu_2 \approx \nu_1$ and $x_2 > x_1$, this Eq.(25) can not be satisfied, and <u>cannot</u> invade. But, if ν_2 is sufficiently larger than ν_1, then the right hand side (RHS) of Eq.(25) gets bigger <u>AND</u> $\left(x_2 - \frac{\nu_2}{\nu_1} x_1\right)$ gets smaller — can even be negative if $\frac{\nu_2}{\nu 1} > \frac{x_2}{x_1}$ — and B_2 <u>CAN</u> invade.

To be more explicit, we need to put in the value of T^* (which, remember, is related to our earlier, RESCALED, T^* by $T^* = \frac{\omega}{y} T'^*$) for the $R - B_1 - T$ system with "$a > 1$" (smaller C values), we have the system stable at $T'^* \approx \frac{b}{a} - 1$, whence $T' \approx (\nu_1/y - \omega)/y$.

So, the condition for B_2 to be <u>UNABLE</u> to invade is (for $a > 1$)

$$x_2 > \frac{y\omega\left(\nu_2 - \nu_1\right)}{\nu_1\left(\frac{\nu_1}{a} - \omega\right)} + \frac{\nu_2}{\nu 1} x_1 \tag{26}$$

For $R - B_1 - T$ with "$a < 1$" let us first look at the corresponding criterion for B_2 unable to invade the stable equilibrium point $R^* - B_1^* - T^*$, and second consider the case when, in practice, we have cycles of large amplitude, weakly damped.
First, for "$a < 1$", $T'^* \approx b - 1 \equiv \frac{\nu_1 - \omega}{\omega}$
This gives the UNABLE to invade criterion as:

$$x_2 > \frac{y\omega\left(\nu_2 - \nu_1\right)}{\nu_1\left(\nu_1 - \omega\right)} + \frac{\nu_2}{\nu_1} x_1 \tag{27}$$

Notice that Eq.(27) has the larger denominator, ($\nu_1 - \omega$), than Eq.(26) when $a > 1$, where the corresponding factor is $\left(\frac{\nu_1}{a} - \omega\right)$, so it is easier to keep B_2 out when $a < 1$.

It is also important to notice also that the long time in which the system is cycling will create conditions in which T will be often (roughly half the time) less than T^*. Even, so, it is likely that Eq.(26) remains a good approximation to the "**e**xclusion criterion" even when we are in the very-weakly-damped cycle phase of the system.

To make things slightly more clear, if we put explicit parameter values into equations Eq.(26) or Eq.(27):

$$\nu_2 \equiv \delta\nu_1 \quad \mathit{with} \quad \delta > 1, \quad \mathit{and} \quad x_2 \equiv \theta x_1, \quad \mathit{with} \quad \theta > 1 \tag{28}$$

And $\nu = 1$; $\omega = 0.1$, $x_1 = 5 \times 10^{-6}$; $y = 4 \times 10^{-10}$, gives

for Eq.(25): $a > 1$ (small C) $\Rightarrow B_2$ cannot invade iff
$\theta > \psi + \left(\frac{4}{5} \times 10^{-5}\right)\left(\frac{\theta - 1}{\frac{1}{a} - 0.1}\right)$ That is, $\theta > \psi$ to approximate neglect 10^{-5}!

For Eq.(26): $a < 1$ (big $C \leftrightarrow$ most of the simulations)
(although complicated by oscillations)
cannot invade *iff* $\theta > \psi + \left(\frac{8}{9} \times 10^{-5}\right)(\psi - 1)$
That is, we can assume the term 10^{-5} as neglectable and the condition again reduces to $\theta > \psi$

Even when more analysis can be done with this system our interest is to show that it is possible to find a condition under which a second species can not invade an established population that produces a self-killing toxin. The existance of such condition opens the gates to additional interesting questions on the way such system works and the toxin is maintained.

6 Toxin sensitivity of other species of *Streptococcus*

As noted in our report, the naturally occurring clinical isolates of *S. pneumoniae* we tested oscillate when put into chemostat culture. Based on the spot assay on lawns, other species of *Streptococcus* are also susceptible to this toxin. To ascertain the relationship between this sensitivity and the genetic relationship of these assayed species we used a maximum likelihood reconstruction protocol for the phylogenetic reconstruction and the parsimony reconstruction of the sensitivity to the toxin overimposed to it.

To ascertain the relationship between toxin sensitivity and the genetic relationship of the assayed *Streptococci* species, we performed a phylogenetic reconstruction of the relationships among species using the small ribosomal sub-unit (16S) genes, available for these species in GenBank. A Maximum likelihood (ML) reconstruction was performed as implemented in PAML 4.0 [1] from a starting Neighbor Joining (NJ) tree obtained in Mega 4.0 [2]. Support for the nodes was inferred by two procedures: i) bootstrap support for the nodes was estimated in multiple ML searches of the trees in PAML 4.0 and applied a 50% majority consensus rule to assign support to the nodes; ii) Bayesian posterior probabilities as implemented in Mr. Bayes 3.4b [3, 4]. Both produced similar results and only the boostrap support is shown in the results. We assayed toxin activity as explained in the materials and methods section of the manuscript, and performed a parsimony reconstruction of toxin sensitivity with Mesquite [5].

The relationships inferred show that *S. mitis* and *S. oralis* are the closest related species to *S. pneumoniae* within the group of Streptococci species considered for the assays (see Figure S6). The colors of the branches correspond to the activity of the supernatant on each one of the terminal species as well as the Maximum parsimony reconstruction of the activity in the deeper nodes (performed in Mesquite, Madison and Maddison 2007). In red it is shown the branches on which activity was detected or inferred, and in black the branches were no activivity was detected or inferred.

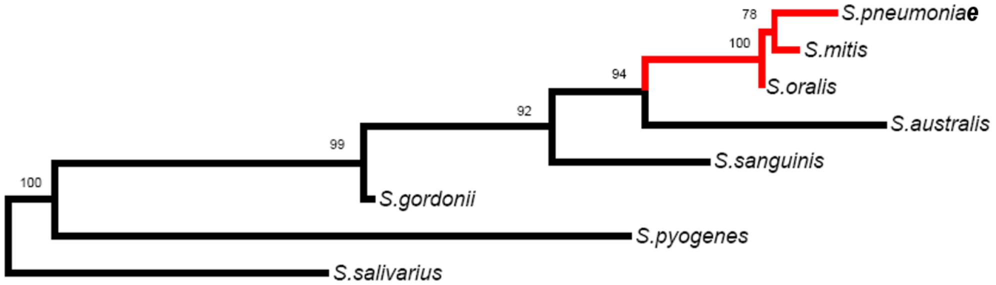

Figure S6: Maximum Likelihood (ML) phylogenetic tree of the Streptococcal species tested for sensitivity to the R6 toxin. Bootstrap support of the nodes for trees based upon 1000 pseudo-replicates, are presented on the top of the nodes. The species in red text are sensitive to the *S. pneumoniae* supernatant. Branches in light red are parsimonious reconstructions of expected activity of the toxin. Distances are measured in units of numbers of substitutions per site.

References

[1] Ziheng Yang. Paml 4: phylogenetic analysis by maximum likelihood. *Mol Biol Evol*, 24(8):1586–1591, Aug 2007. doi: 10.1093/molbev/msm088. URL http://dx.doi.org/10.1093/molbev/msm088.

[2] Koichiro Tamura, Joel Dudley, Masatoshi Nei, and Sudhir Kumar. Mega4: Molecular evolutionary genetics analysis (mega) software version 4.0. *Mol Biol Evol*, 24(8):1596–1599, Aug 2007. doi: 10.1093/molbev/msm092. URL http://dx.doi.org/10.1093/molbev/msm092.

[3] J. P. Huelsenbeck and F. Ronquist. Mrbayes: Bayesian inference of phylogenetic trees. *Bioinformatics*, 17(8):754–755, Aug 2001.

[4] Fredrik Ronquist and John P Huelsenbeck. Mrbayes 3: Bayesian phylogenetic inference under mixed models. *Bioinformatics*, 19(12):1572–1574, Aug 2003.

[5] W.P Maddison and D.R. Maddison. Mesquite: a modular system for evolutionary analysis. version 2.0, 2007. URL http://mesquiteproject.org.
